# Molecular Mechanisms Associated with the Development of the Metritis Complex in Dairy Cattle

**DOI:** 10.3390/genes15040439

**Published:** 2024-03-30

**Authors:** Leanna Sanchez, Fernando Campos-Chillon, Mehdi Sargolzaei, Daniel G. Peterson, Kim A. Sprayberry, Garry McArthur, Paul Anderson, Bruce Golden, Siroj Pokharel, Mohammed K. Abo-Ismail

**Affiliations:** 1 Department of Animal Science, California Polytechnic State University, 1 Grand Ave., San Luis Obispo, CA 93407, USA; sanch49@calpoly.edu (L.S.); lcamposc@calpoly.edu (F.C.-C.); dpeterso@calpoly.edu (D.G.P.); kspraybe@calpoly.edu (K.A.S.); spokhare@calpoly.edu (S.P.); 2 Select Sires Inc., 11740 US-42, Plain City, OH 43064, USA; msargol@uoguelph.ca; 3 Center for Genetic Improvement of Livestock, University of Guelph, Guelph, ON N1G 2W1, Canada; 4 Swinging Udders Veterinary Services, 8418 Liberty Rd, Galt, CA 95632, USA; swingingudder@gmail.com; 5 Department of Computer Science and Software Engineering, California Polytechnic State University, 1 Grand Ave., San Luis Obispo, CA 93407, USA; pander14@calpoly.edu; 6 Theta Solutions LLC, Olympia, WA, USA; blgolden57@gmail.com

**Keywords:** genome-wide association study, metritis complex, dairy cattle

## Abstract

The metritis complex (MC), a group of post-partum uterine diseases, is associated with increased treatment costs and reduced milk yield and fertility. The goal of this study was to identify genetic variants, genes, or genomic regions that modulate MC disease. A genome-wide association study was performed using a single-locus mixed linear model of 1967 genotypes (624,460 SNPs) and metritis complex records. Then, in-silico functional analyses were performed to detect biological mechanisms and pathways associated with the development of MC. The *ATP8A2*, *COX16*, *AMN*, and *TRAF3* genes, located on chromosomes 12, 10, and 21, were associated with MC at *p* ≤ 0.0001. These genes are involved in the regulation of cholesterol metabolism in the stromal tissue of the uterus, which can be directly associated with the mode of transmission for pathogens causing the metritis complex. The modulation of cholesterol abundance alters the efficiency of virulence factors and may affect the susceptibility of the host to infection. The *SIPA1L1*, *DEPDC5*, and *RNF122* genes were also significantly associated with MC at *p* ≤ 0.0001 and are involved in the PI3k-Akt pathway, responsible for activating the autophagic processes. Thus, the dysregulation of these genes allows for unhindered bacterial invasion, replication, and survival within the endometrium.

## 1. Introduction

During the peripartum period, dairy cows are particularly vulnerable to uterine infection caused by pathogenic bacteria including *Escherichia coli*, *Fusobacterium necrophorum*, and *Trueperella pyogenes*, resulting in a significant increase in the prevalence of uterine diseases [1,2]. The metritis complex refers to a persistent group of post-partum uterine diseases that affect 20–40% of dairy herds, with a 25% average incidence rate per herd [3], and include all forms of endometritis, metritis, and pyometra diagnosed after parturition. These diseases result in an annual cost of approximately USD 900 million to the US dairy industry due to their high prevalence, treatment cost, and negative impact on milk production, uterine functionality, and reproductive efficiency [4,5,6,7]. These infections often follow placental retention, abortion, and dystocia, and are accompanied by clinical signs of pyrexia, loss of appetite, depression, and reduced feed intake [1,7]. In an effort to improve diagnostic accuracy, clinical endometritis (an infection of the endometrial tissue producing purulent or mucopurulent discharge), metritis (fetid reddish-brown discharge), and pyometra (pus accumulation in the uterus concomitant with a corpus luteum) have been consolidated into a single category, defined as the metritis complex (MC), due to the variations in clinical diagnosis and often ambiguous commercial dairy operations records [8].

Recent years have seen an increase in studies focused on genomic selection for calving ease, fertility, and health traits, leading to an increase in the genetic trends of functional traits, and subsequently bringing to light the negative impacts of MC in dairy cattle [9]. Health traits, such as the development of MC, have been found to be highly complex due to their complex genetic architecture [10,11]. In addition, these traits exhibit lower genomic heritability due to high environmental variance, making it difficult to detect genetic variants and genes prompting variations among animals in these traits. Nonetheless, with the advent of high throughput sequencing and genotyping, genome-wide association studies (GWASs) have been a powerful tool to identify genes for common diseases and quantitative traits [12]. Recent studies using single-nucleotide polymorphism (SNP) data estimated the heritability of metritis from 0.004 to 0.07 [13,14,15], indicating that genetic variance could be captured [14,16]. This does not mean that MC is not controlled by genes, but the genomic variances caused by genes are relatively small compared to the environmental variance. If genetic variation is detected for uterine disease traits, this allows for genetic progress at a slow rate and the detection of genes or molecular mechanisms with strong effects on these traits [15]. Thus, a few GWASs have successfully identified genetic variants (e.g., single-nucleotide polymorphisms (SNPs)) involved in the genetic basis of health traits in cattle using small to medium-density SNP panels [13,15,17]. Potential SNPs and genes on chromosomes BTA 1, 2, and 21 were associated with endometritis in German Holstein cows [17]. Recently, another GWAS on German Holstein Friesian cattle revealed 24 potential genes for all uterine disease traits [13]. Another GWAS discovered 51 genes located on nine different chromosomes that are associated with metritis in Canadian Holstein cows [15]. Nonetheless, causative variants for MC remain relatively unknown, and missing heritability could be claimed [18]. In addition, there is a need to detect genes and pathways from commercial dairy cattle that could be validated on the same herds using integrated omics approaches such as metabolomics and proteomics. It is also important to validate previously reported genes and genomic regions for uterine disease traits using higher-density SNP panels—used in the current study—that can be used in genomic selection. Genomic selection offers a promising opportunity to improve health traits including MC. Therefore, the objectives of this study were to (1) estimate genomic heritability for MC using HD SNP panels, (2) identify genetic variants and genes associated with the development of MC via a GWAS using high-density chip panels (HD SNPs), and (3) detect the biological mechanisms or pathways associated with MC development.

## 2. Materials and Methods

### 2.1. Animals and Phenotypic Data

This study involved Holsteins and Jersey breeds at three dairy operations: Herd 1 (located in the coastal region of central California) and Herds 2 and 3 (located in central California). We extracted the historical records of primi- and multiparous dairy cattle born between 2015 and 2021 from a complied producer-recorded dataset of over 17,000 animals using DairyComp 305^TM^ Herd management software Release 27 (Valley Agricultural Software, Tulare, CA)). For the purpose of this study, diagnosed cases of metritis, endometritis, or pyometra within the transition period were collectively grouped as the phenotype referred to as the metritis complex (MC) and designated as cases [7,19,20]. Thus, MC includes the diseases defined as clinical metritis, clinical endometritis, and pyometra, as described by Sandals et al. (1979) [8]. All diseases were diagnosed by a trained herdsman or veterinarian. In the case of one or multiple documented MC diagnoses, these cows were assigned as cases with MC. All cows without a diagnosis of MC or any other disease were assigned as controls (i.e., healthy).

### 2.2. DNA Extraction and Genotypic Data

A total of 2120 cows were sampled using different approaches. Blood collected from the coccygeal vein (*n* = 679) and ear notch samples (n = 1441) were sent for DNA extraction using the standard commercial kit and sequenced for low-pass skim sequencing by NEOGEN^®^ Genomics. The genomic coverage was from 0.5 to 3.0× and the concordance averaged 99.3% in genotype concordance to the illumina SNP chips [21]. The assessment of imputation accuracies from low-pass sequencing to commercial SNP panels was performed using the Gencove pipeline [21] adopted by NEOGEN^®^ Genomics. For this study, the bovine high-density (HD; 782,138 SNPs) panel genotypes were extracted and utilized for GWAS analyses. Quality control was applied to genotypes using the PLINK v1.9 [22] software. Genetic variants with a minor allele frequency (MAF) of less than 0.01 and those deviating from the Hardy-Weinberg equilibrium were removed. Additionally, animals with duplicate genotypes and breeds other than Holstein and Jersey were excluded from the analysis. The final dataset included 624,460 SNPs and a total of 1967 cows consisting of 442 cases with MC and 1525 healthy controls.

### 2.3. Population Structure

To assess population stratification, a multidimensional scaling (MDS) analysis based on genome-wide identity-by-state pairwise distances was performed using PLINK v1.9 [22]. Linkage disequilibrium pruning was also conducted to reduce the likelihood of obtaining principal components based on a few genomic regions, and the analysis was carried out with 66,245 genetic variants. The MDS plot was visualized using “scatterplot3d” and “ggplot2” in R [23,24,25].

### 2.4. Genome-Wide Association Study (GWAS) and Genomic Heritability

The GWAS analysis was conducted on animals (n = 1967) with genotypes (624,460 SNPs) and phenotypes that passed the quality control. Variance components and genomic heritability were estimated using the genomic best linear unbiased prediction (gBLUP) statistical method implemented in the Genome-wide Complex Trait Analysis (GCTA) software version 1.94.1 [26] through a restricted maximum likelihood algorithm (REML). To perform the GWAS, a single-SNP mixed linear animal model was constructed using GCTA software. The allele substitution effect and the association level of significance were estimated. The model included the fixed effects of breed, herd, and SNP and utilized the genomic relationships of all animals. The model is represented as follows:

(1) $$Y_{\mathrm{ijk}}=\mu +\beta_{1}\mathrm{SNP}+{\mathrm{Breed}}_{i}+{\mathrm{Herd}}_{j}+a_{k}+e_{\mathrm{ijk}}$$

where Y_ijk_ is the diagnosis of MC (i.e., phenotypes) in the kth animal of the ith breed, µ is the overall mean for the trait, Herd_j_ is the fixed effect of the *j*th herd, Breed_i_ is the fixed effect of the jth breed, β_1_ is the allele substitution effect of the candidate SNP being tested, SNP represents the SNP genotype variable (coded as 0, 1, or 2), a_k_ is the random additive genetic (polygenic) effect of the kth animal, and e_jklm_ is the residual random effect associated with the kth animal record. Assumptions for this model were a_k_: a~N (0, G σ^2^a), where G is a genomic relationship matrix and σ^2^a is the additive genetic variance, and e_ijk_: e~(0, I σ^2^e), where I is the identity matrix and σ^2^e is the error variance. The expectations were that E(a_k_) = 0 and E(e_ijk_) = 0, and the variances were Var(a_k_) = σ^2^a and Var(e_ijk_) = σ^2^e. Gσ^2^a is the covariance matrix of the vector of genomic additive genetic effects and the genomic relationship matrix (G).

To declare a significant association between an SNP and MC, we considered a suggestive significance candidate threshold (*p*-value = 1 × 10^−4^) [13,27,28]. We also identified the top 100 significant (*p* ≤ 0.00025) SNPs for further functional analyses as the genomic inflation factor indicated a slight underestimation of *p*-values. We used a less stringent *p*-value threshold to declare the significance associated with MC for two main reasons: (1) the known polygenic nature of health traits and (2) to increase the power of quantitative trait loci (QTL) detection (e.g., genetic variants/genes/genomic region) with small but true effects on MC development. In addition, relaxing the threshold for declaring significant association is consistent with previous publications on GWASs for fertility and health traits in dairy cattle using SNP panels [29,30]. To visualize the GWAS results, a Manhattan plot and Quantile–Quantile (Q-Q) plot were generated using the R package “qqman” [31]. The Q-Q plot and the genomic inflation factor were used to evaluate the bias in the estimated *p*-values from the association analyses. The genomic heritability of MC was estimated using the additive genomic variance divided by the total phenotypic variance calculated via REML methods implemented in GCTA [26]. The proportion of genetic variance explained by the top 100 SNPs for MC was estimated using GVCBLUP software version 3.9 [32].

### 2.5. In-Silico Functional Analyses

The SNPs significantly (*p* ≤ 0.00025) associated with MC were mapped to the bovine genome ARS-UCD1.2 assembly using the BioMart tool in the Ensembl database [33] to identify the nearby gene(s) located within 10 kilobases upstream or downstream. Enrichment analyses of Kyoto Encyclopedia of Genes and Genomes (KEGG) pathways and gene ontology (GO) terms were performed using the Database for Annotation, Visualization, and Integrated Discovery (DAVID) bioinformatics tool [34]. To investigate if any of the identified candidate genes in the current study had previously been reported for QTLs related to uterine infection, the Cattle QTL database Release 50 [35] was accessed on 18 April 2023.

## 3. Results

The descriptive statistics of the observed phenotypes and incidence rates separated by herd are presented in Table 1. A total of 1967 out of 2124 multiparous cows were used in this study after removing duplicate animals, low-quality genotypes, and other breeds. The records for Jersey (13.5%) were fewer than those of the Holstein (86.5%) breed. Nonetheless, Jersey (22.8%) and Holstein displayed similar diseased phenotype diagnoses, with 22.8% and 21.8% for Holstein and Jersey, respectively. Of the three herds, the incidence rate of cattle diagnosed with a uterine disease within the metritis complex (endometritis, metritis, and pyometra) was 33.7%, 16.1%, and 24.5% with respect to Herds 1, 2, and 3.

### 3.1. Population Structure

**Figure 1:**
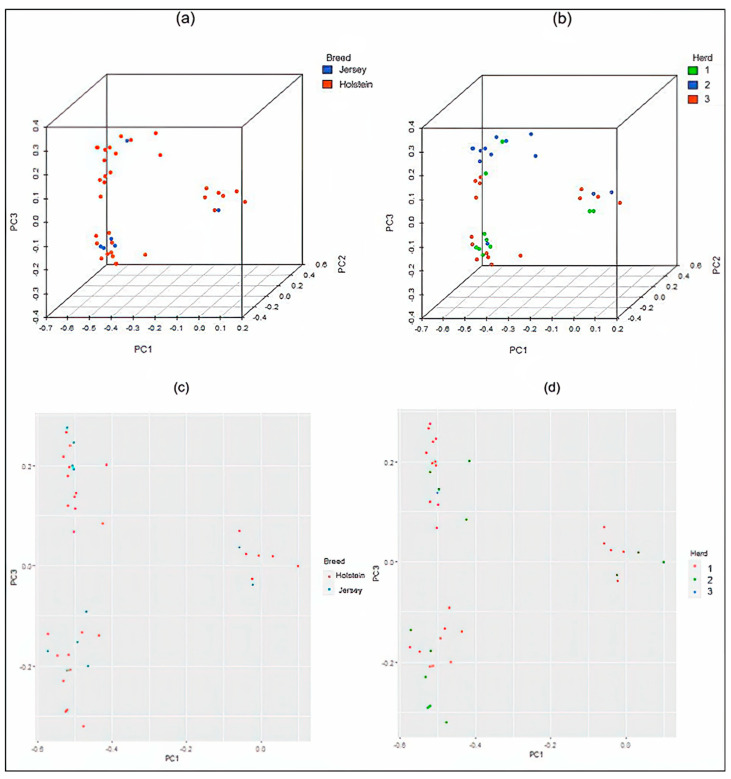
The multidimensional scaling (MDS) analysis. Representative of the 624,460 single-nucleotide polymorphisms (SNPs) and 1967 dairy cows separated according to (**a**) breed and (**b**) herd. The 3D format shows the population structure of the first three principal components (PCs), representing the distribution of genetic variation across breeds and herds. The 2D format shows a visualization for (**c**) breed and (**d**) herd between PC1 and PC3.

The MDS analysis, which is similar conceptually to principal component analysis, revealed no stratification due to herd and breed, as illustrated in Figure 1a,b. The results from plotting PC1 against PC3 showed no difference in genetic structure stratification, illustrated in Figure 1c,d. Nonetheless, to account for the slight stratification in family structure, shown in Figure 1, we included the cluster groups obtained by MDS analysis as fixed effects in the MLM model, but this caused a severe underestimation of observed *p*-values in comparison with the expected *p*-values. In addition, the genomic inflation factor from the model with MDS decreased, indicating an underestimation of SNP effects and overparameterization of the model. Thus, the cluster groups were eliminated from the model in further analyses. The value of the genomic inflation factors from the GWAS evaluated models ranged between 0.97 and 0.999, indicating the absence of bias in the estimated SNP effects or *p*-values due to family structure.

### 3.2. Genome-Wide Association Study (GWAS) and Genomic Heritability

**Figure 2:**
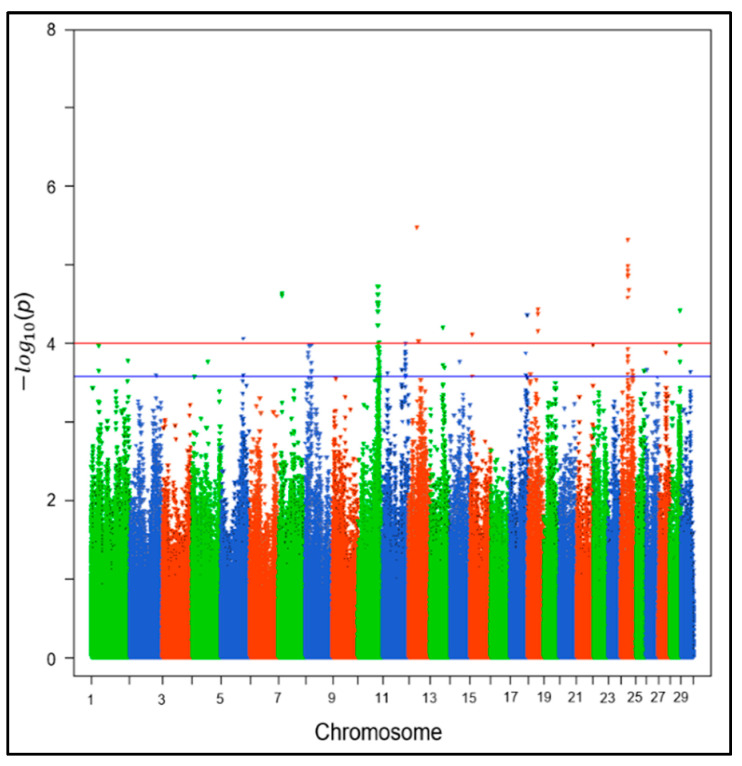
Genome-wide association study (GWAS) results from 624,460 SNPs and 1967 dairy cattle across three herds diagnosed with at least one disease in the metritis complex. Manhattan plot for −log10 *p*-values of SNP effects for the metritis complex in Holstein and Jersey dairy cows. The red horizontal line corresponds to a *p*-value threshold of −log10 ≥ 4.00. The blue line denotes the greatest *p*-value within the top 100 significant single-nucleotide polymorphisms (SNPs) at −log10 = 3.59.

The results from the GWAS, presented as a Manhattan plot displaying the −log10 *p*-value of each polymorphism with respect to their autosomal positions, for the metritis complex are illustrated in Figure 2. In this study, the genomic heritability was 0.04 (±0.02) and the calculated genomic inflation factor (λ) was 0.98. The Q-Q plot confirmed the findings from the genomic inflation factor, with a slight underestimation of *p*-values (Supplementary Figure S1).

The top ten most significant SNPs were located on Bos taurus autosome (BTA) 24, 10, 12, and 7 with 6, 2, and 1 significant SNP(s), respectively. The SNPs most significantly associated with the metritis complex were rs133231370, rs109438305, rs109839478, rs42606152, rs42606142, rs42606134, rs137544510, rs110717900, rs134298785, and rs134805934. Of the top 100 significant SNPs, most of the associations were identified on BTA 10 (n = 34), BTA 11 (n = 18), and BTA 24 (n = 13). We identified 40 SNPs within the flanking or transcribed regions of 20 potential candidate genes, listed in Supplementary Table S1. These genes were located on BTA 4, 8, 10, 11, 12, 15, 17, 18, 21, 25, and 27, with BTA 10 being the most associated region, containing 6 of the 20 genes.

The region around 8,150,383–81,931,753 bp on BTA 10 held three genes: Solute Carrier Family 10 Member A1 (*SLC10A1*), Serine and Arginine-rich Splicing Factor 5 (*SRSF5*), and Solute Carrier Family 8 Member A3 (*SLC8A3*). Along with this, the regions around 10,026,821 bp and 29,252,315 bp on BTA 18 and 27, respectively, were linked to two genes: Cadherin-13 (*CDH13*) and Ring Finger Protein (*RNF122*). These regions on BTA 10, 18, and 27 have previously been reported as QTLs related to dystocia in Holstein cattle. The regions around 2,461,770 bp, 83,245,103 bp, 82,066,559 bp, and 92,480,213 bp on BTA 8 (n = 1), BTA 10 (n = 2), and BTA 11 (n = 1), respectively, were linked to four genes: Solute Carrier Family 24 Member A2 (*SLC24A2*), Cytochrome C Oxidase Assembly Factor (*COX16*), Signal Induced Proliferation Associated1 Like 1 (*SIPA1L1*), and DAB2 Interacting Protein (*DAB2IP*), all reported regions containing previous QTLs associated with stillbirth. We identified regions around 66,416,290 bp, 65,237,605 bp, and 34,170,987 bp on BTA 4, 17, and 25, respectively, linked to four genes: WAS/WASL Interacting Protein Family Member 3 (*WIPF3*), Secerin-1 (*SCRN1*), Uncharacterized Protein KIAA1671 (*KIAA1671*), and Cytochrome p450 Oxidoreductase (*POR*), each of which is QTLs previously reported relating to calving ease in dairy cows. The regions around 33,569,134 bp, 8,809,053 bp, and 70,481,417 bp on BTA 12, BTA 15, and BTA 17, respectively, were linked to three genes: ATPase Phospholipid Transporting 8A2 (*ATP8A2*), Contactin-5 (*CNTN5*) Signal, and DEP Domain-containing 5 Protein (*DEPDC5*), reported previous as QTLs associated with non-return rates in dairy cows. Ten significant SNPs spanning 87,619,311–87,650,109 on BTA 10 corresponded to one gene, Estrogen-related Receptor β (*ESRRB*), and this location housed a primary QTL for the occurrence of placental retention in the post-calving period. The remaining SNPs, rs110783124 and rs135585624, were not previously reported to any fertility QTL specific to Holstein or Jersey cattle.

### 3.3. In-Silico Functional Analyses

The enrichment analyses for gene ontology and the KEGG pathway for the 20 identified genes within or flanking the regions associated with the metritis complex were not significant at a 5% false discovery rate. Nonetheless, GO terms Regulation of Dendrite Morphogenesis, Regulation of Axonogenesis, Ephrin Receptor Signaling Pathway, Actin Cytoskeleton Reorganization, and GTPase Activator Activity were enriched at *p* < 0.05, (Supplementary Tables S2–S5). The summary of all GO terms and KEGG pathways linked to the gene list associated with the metritis complex are given in Supplementary Tables S2–S5.

## 4. Discussion

Diseases such as metritis, endometritis, and pyometra have been the topic of numerous studies within the last decade due to their negative effects on animal welfare, animal efficiency, and the economic welfare of the dairy industry [36,37,38]. Thus, one of the main goals of the current study was to understand the genetic architecture of uterine disease diagnoses in the post-partum period. In the present study, the estimated genomic heritability was 0.04 for the metritis complex. The proportion of genetic variance explained by the top 100 SNPs for MC was 0.1792 ± 0.04. While some studies have reported pedigree-based heritability of metritis, there are few published estimates for genomic or SNP-based heritability for MC. A previous study using imputed and actual 44,747 genotypes and a linear model estimated genomic heritability as 0.008, 0.01, and 0.004 for metritis, endometritis (mucopurulent), and pyometra, respectively, and gave an estimate of 0.02 when assessing the genomic heritability of the metritis complex [13]. Their genomic heritability was smaller than what was reported based on pedigree-based estimates due to the inability of the imputed 50K SNP chips to fully capture the genetic variation in the whole genome [13]. In the current study, we utilized high-density SNP chips (624,460 SNPs), which resulted in similar heritability to that when using pedigree-based analysis with first-parity records but slightly smaller heritability than that with genomic-based analysis (h^2^ = 0.06–0.07) reported from producer-recorded metritis event data in US dairy cattle [14]. Thus, these results support that genomic improvement via selection for MC is feasible in the United States using producer-recorded data and combining uterine diseases as part of the MC [14].

In this genome-wide association study, the top 100 significant SNPs were within or flanking 20 genes. These genomic regions were previously reported to be associated with other QTLs, including placenta retention (n = 1), calving ease (n = 4), stillbirth (n = 4), and dystocia (n = 5), and each has also been reported for their roles in the development of uterine infection or because of uterine infection in dairy cattle. With this, as many as 54.8% of cows that have placental retention are soon diagnosed with at least one of the diseases that are part of the metritis complex, and dystocia was seen preceding 40%, 48%, and 10%, of metritis, endometritis, and pyometra cases, respectively [39,40]. Here, calving ease, or the scale of calving difficulty experienced during parturition, is interchangeable with the true definition of dystocia and closely related to stillbirth [41]. The incidence of stillbirth has been previously associated with the pathogenic invasion of the maternal and fetal tissues by *Arcanobacterium pyogenes* and *E. coli*, two prominent bacterial species closely tied to the development of metritis and endometritis [1,42]. Further, three QTLs for non-return rates, the proportion of cattle that is not re-bred within a certain time frame proceeding insemination, corresponded to the three separate significant regions on autosomes 12, 15, and 17, suggesting their relevance in post-partum uterine disease traits [43]. Metritis has been found to impede ovarian functionality, prolong ovulatory periods, and disrupt breeding timeframes, thereby contributing to an increased non-return rate [44]. Interestingly, cows diagnosed with placental retention and/or a disease within the metritis complex have a reduced likelihood of becoming pregnant post-artificial insemination and a heightened chance of pregnancy loss [45]. With this, a transparent linkage is seen between multiple uterine infections, the precursory complications in the antepartum to post-partum period, and their resulting impact on future fertility traits, forming a multiplex of reproductive setbacks and economic deficiencies. This transpicuous linkage shown between the recognized QTLs and uterine disease in dairy cattle validates the power of our GWAS and its ability to identify potential genes and genetic variants associated with the metritis complex. The current findings confirmed how complications throughout pregnancy increase vulnerabilities within the vaginal canal, cervix, placenta, and endometrium and establish an environment destined for infection [46,47].

The most significant SNP, rs133231370, corresponded to a nearby hub gene, *ATP8A2*, known for its involvement in lipid metabolism. Previously, *ATP8A2* has been discussed for its role in milk fat synthesis in Holstein cattle [48]. This gene produces an ATPase protein responsible for transferring, or flipping, phosphatidylserine (PS) and phosphatidylethanolamine from the ectoplasmic to the cytoplasmic layers of the cell membrane lipid bilayer. The nature of this function results in the asymmetric partitioning of lipids across the membrane, which is vital for vesicle trafficking, cell signaling, apoptosis, cell survival, and cholesterol/bile homeostasis [49]. The *ATP8A2* gene is also expressed within the uterus, where it plays a role in lipid metabolism and cholesterol abundance. Interestingly, higher cholesterol abundance within the uterine endometrial tissue increases pathogenic invasion through pyolysins, cholesterol-dependent cytolysins from *T. pyogenes*, a prominent Gram-positive bacteria in purulent infections, such as the metritis complex [50].

Importantly in the current study, five other genes expressed within the uterus corresponding to significant SNPs (*SLC10A1*, *COX16*, *AMN*, *TRAF3*, and *POR)* were found to regulate cholesterol levels. The *TRAF3* and *COX16* genes work to regulate intracellular cholesterol indirectly through their involvement in neighboring pathways. The *TRAF3* gene, an immunity-related gene in cattle, was previously found to be a negative regulator of the nuclear factor-κB (NF-κB) pathway, a pathway known to increase cholesterol accumulation within the cell to promote atherosclerosis and macrophage foam cell formation in recent culture studies [51,52]. Similarly, *COX16* has been shown to inhibit the signaling activity of p53, which cooperates with the Hippo pathway to regulate the downstream activity of the sterol regulatory element-binding proteins (SREBPs; [53,54]). These proteins activate a multitude of genes responsible for the uptake and metabolism of phospholipids, triglycerides, fatty acids, and, cholesterol [55]. Conversely, the activity of *SLC10A1*, *AMN*, and *POR* works directly to alter cholesterol levels within the endometrium. The *POR* gene encodes the cytochrome p450 protein, previously seen in differing levels between healthy and metritis cows, and is known to produce superoxides during the oxidation process, contributing to the reactive oxygen species (ROS) pool [56,57]. This ROS activity increases cholesterol and glucose influx and the synthesis of cholesterol from glucose [58]. Likewise, *SLC10A1*, once translated, produces a protein that co-transports sodium and bile acids, the catabolic product of cholesterol, in the dairy cow post-partum endometrium, showing the importance of *SLC10A1* in intracellular cholesterol homeostasis [59,60]. The *AMN* gene, found to be expressed within the cattle uterine epithelial tissue at all stages of estrous, encodes the amnionless protein, which functions to anchor another protein, cubilin, to the membrane [61]. Cubilin is an endocytic lipoprotein receptor known to mediate high-density lipoprotein (HDL) cholesterol endocytosis [62]. Our study observed a functional relationship between the genes corresponding to SNPs significantly associated with the metritis complex in dairy cows and the importance of cholesterol regulation in uterine tissues. This study confirms that there is crosstalk between cholesterol abundance, pathogenic invasion, and susceptibility to uterine infection; however, details should be further elucidated [50,63].

Moreover, in the current study, 9 of the 20 genes found flanking the top significant SNPs were discovered to be linked to the phosphatidylinositol-3-kinase/protein kinase B (PI_3_K/Akt) pathway. The PI_3_K/Akt pathway has been recently discussed in terms of its essential role in signal transduction, cell proliferation, apoptosis, and autophagy and its correspondence to pathogenesis within bovine endometrial epithelial cells [64]. The activated pathway phosphorylates Akt downstream, and active Akt has been found to deter the fusion of the autophagosome to the lysosome, allowing for the survival of pathogenic materials and promoting infection [65,66]. The *SIPA1L1*, *DEPDC5*, and *RNF122* genes encode proteins that repress the RAS, mTOR, and Rig-1 signaling pathways, respectively [67,68,69]. These secondary pathways work to inhibit, activate, and compete with the activity of PI_3_K/Akt and, consequently, regulate autophagosome elongation, maturation, and termination and the autophagy process [70,71,72]. The PI_3_K/Akt pathway was considerably discussed for its role in bovine physiology including oocyte competence, immune responsiveness, milk fat synthesis, bacterial resistance, vascular homeostasis, and angiogenesis [64,73,74,75,76]. Our study reports the link between the development of uterine infections in dairy cattle and the PI_3_K/Akt pathway, considering its major role in pathogenesis and the immune response. Little is known about the biological function of the remainder of the identified genes, *CNTN5*, *SRSF5*, *SLC24A2*, *SLC8A3*, and *KIAA1671*, regarding *B. taurus*.

This study revealed unique genetic variants and corresponding candidate genes associated with the metritis complex. This study acknowledges that there is no advantage in accuracy when using an HD SNP panel compared to that of a medium-density panel for the GBLUP method in Holstein and Jersey cows. With this, the power of associated polymorphism detection may be affected by including data from Jersey breeds compared to those of the Holstein breed, given that detecting QTLs for a multi-breed population requires a larger sample population size [77]. Nonetheless, utilizing a multi-breed population for reference permits an increase in the accuracy of genomic estimated breeding values (GEBVs) for smaller breeds and the potential use of the detected genetic variant for a crossbreed genomic evaluation.

Previously identified fertility-related genes, such as *POR* and *SCRN1*, were also revealed in this study as candidate genes corresponding to uterine disease in the transition period [57,78]. A number of the putative genes for the metritis complex have already been associated with immunity and reproductive efficiency in past publications [79,80]. The underlying common biological processes amongst the identified candidate genes were further confirmed using the correspondence of past literature in both human and bovine disease studies. As the metritis complex is a complex polygenic trait and develops as a collective infection involving the detriment of multiple species, studies looking to further analyze how these candidate genes may influence the ability of dairy cows to resist infection are required. In addition, an unrelated population is required to validate the detected significantly associated SNPs and genes from this study. Furthermore, these SNPs must be tested for genomic prediction prior to application in breeding programs. In this case, the Jersey breed should be analyzed in a larger sample size to corroborate previous results. The novel SNPs and their corresponding genes identified in this study should be considered candidates associated with the development of uterine disease in dairy cattle. Even so, these results and genetic variants should be further studied in regard to their influence on bacterial resistance and susceptibility to diseases defined under the metritis complex in the transition period. However, future studies using larger sample populations and more dense genotypes (e.g., sequence genotypes with millions of genetic variants) may increase the resolution of detecting the causal mutation and candidate genes for the metritis complex. Further validation studies are required to confirm the detected genetic variants and genes before use in genomic prediction for the metritis complex.

## 5. Conclusions

This study provided a novel perspective on the genetic architecture of uterine infection, identifying genetic variations and genes associated with the metritis complex in Jersey and Holstein cattle. We detected 40 SNPs and 20 candidate genes flanking the significant genomic regions. Several genes expressed in the reproductive tract were found to be linked to two processes in the endometrial tissue: the PI_3_K/Akt pathway and cholesterol homeostasis. These findings provide new insights into the proportion of genetic variability elucidated by SNPs for uterine disease and shed light on the physiological vulnerabilities that can lead to the pathogenesis of metritis, endometritis, and pyometra after calving. The results of this study should be taken into consideration when selecting index Holstein and Jersey dairy cattle.

## Figures and Tables

**Table 1:** Descriptive statistics for the metritis complex in Holstein and Jersey multiparous cows.

Herd	Sample	Breed		Phenotype ^1^	
	n	Jersey	Holstein	Case	Control
1	525	262	263	171	354
2	980	4	976	158	822
3	462	0	462	113	349
Total	1967	266	1701	442	1525

^1^ Case = the diagnosis of at least one disease from the metritis complex; Control = no prior diagnosis from the metritis complex.

## Data Availability

Genotype datasets cannot be publicly shared due to intellectual property issues. Other datasets from this study can be provided by email upon request.

## Supplementary Materials

The following supporting information can be downloaded at https://www.mdpi.com/article/10.3390/genes15040439/s1: Figure S1: Quantile–Quantile (QQ) plot of the expected vs. the observed null distribution of the *p*-values for the association between the 624,460 SNPs and 1967 dairy cattle across three herds diagnosed with at least one disease in the metritis complex. Table S1: Identified single nucleotide polymorphisms (SNPs) with flanking candidate genes and associated quantitative trait loci (QTL). Table S2: Enriched gene ontology (GO) biological process (BP) terms for the metritis complex in Jersey and Holstein dairy cattle. Table S3. Enriched gene ontology (GO) cellular component (CC) terms for the metritis complex in Jersey and Holstein dairy cattle. Table S4. Enriched gene ontology (GO) molecular function (MF) terms for the metritis complex in Jersey and Holstein dairy cattle. Table S5. Enriched Kyoto Encyclopedia of Genes and Genomes (KEGG) pathways for the metritis complex in Jersey and Holstein dairy cattle.

## References

[1] Sheldon I.M., Williams E.J., Miller A.N.A., Nash D.M., Herath S. (2008). Uterine Diseases in Cattle after Parturition. Vet. J..

[2] Basbas C., Garzon A., Silva-del-Rio N., Byrne B.A., Karle B., Aly S.S., Champagne J.D., Williams D.R., Lima F.S., Machado V.S. (2022). Evaluation of Antimicrobial Resistance and Risk Factors for Recovery of Intrauterine Escherichia Coli from Cows with Metritis on California Commercial Dairy Farms. Sci. Rep..

[3] Pérez-Báez J., Silva T.V., Risco C.A., Chebel R.C., Cunha F., De Vries A., Santos J.E.P., Lima F.S., Pinedo P., Schuenemann G.M. (2021). The Economic Cost of Metritis in Dairy Herds. J. Dairy Sci..

[4] Figueiredo C.C., Merenda V.R., de Oliveira E.B., Lima F.S., Chebel R.C., Galvão K.N., Santos J.E.P., Bisinotto R.S. (2021). Failure of Clinical Cure in Dairy Cows Treated for Metritis Is Associated with Reduced Productive and Reproductive Performance. J. Dairy Sci..

[5] Giuliodori M.J., Magnasco R.P., Becu-Villalobos D., Lacau-Mengido I.M., Risco C.A., de la Sota R.L. (2013). Metritis in Dairy Cows: Risk Factors and Reproductive Performance. J. Dairy Sci..

[6] Overton M., Fetrow J. Economics of Postpartum Uterine Health. Proceedings of the Dairy Cattle Reproduction Council Convention Annual Meet.

[7] Sheldon I.M., Cronin J., Goetze L., Donofrio G., Schuberth H.-J. (2009). Defining Postpartum Uterine Disease and the Mechanisms of Infection and Immunity in the Female Reproductive Tract in Cattle1. Biol. Reprod..

[8] Sandals W.C.D., Curtis R.A., Cote J.F., Martin S.W. (1979). The Effect of Retained Placenta and Metritis Complex on Reproductive Performance in Dairy Cattle A Case Control Study. Can. Vet. J..

[9] Egger-Danner C., Cole J.B., Pryce J.E., Gengler N., Heringstad B., Bradley A., Stock K.F. (2015). Invited Review: Overview of New Traits and Phenotyping Strategies in Dairy Cattle with a Focus on Functional Traits. Animal.

[10] Freebern E., Santos D.J.A., Fang L., Jiang J., Parker Gaddis K.L., Liu G.E., VanRaden P.M., Maltecca C., Cole J.B., Ma L. (2020). GWAS and Fine-Mapping of Livability and Six Disease Traits in Holstein Cattle. BMC Genom..

[11] Schaid D.J., Chen W., Larson N.B. (2018). From Genome-Wide Associations to Candidate Causal Variants by Statistical Fine-Mapping. Nat. Rev. Genet..

[12] Hirschhorn J.N., Daly M.J. (2005). Genome-Wide Association Studies for Common Diseases and Complex Traits. Nat. Rev. Genet..

[13] May K., Sames L., Scheper C., König S. (2022). Genomic Loci and Genetic Parameters for Uterine Diseases in First-Parity Holstein Cows and Associations with Milk Production and Fertility. J. Dairy Sci..

[14] Parker Gaddis K.L., Cole J.B., Clay J.S., Maltecca C. (2014). Genomic Selection for Producer-Recorded Health Event Data in US Dairy Cattle. J. Dairy Sci..

[15] Guarini A.R., Lourenco D.A.L., Brito L.F., Sargolzaei M., Baes C.F., Miglior F., Misztal I., Schenkel F.S. (2019). Genetics and Genomics of Reproductive Disorders in Canadian Holstein Cattle. J. Dairy Sci..

[16] Weller J.I., Ezra E., van Straten M. (2019). Genetic and Environmental Analysis of Diseases with Major Economic Impact in Israeli Holsteins. J. Dairy Sci..

[17] Naderi S., Bohlouli M., Yin T., König S. (2018). Genomic Breeding Values, SNP Effects and Gene Identification for Disease Traits in Cow Training Sets. Anim. Genet..

[18] Zaitlen N., Kraft P. (2012). Heritability in the Genome-Wide Association Era. Hum. Genet..

[19] Sheldon I.M., Noakes D.E., Parkinson T.J. (2019). The Metritis Complex in Cattle. Veterinary Reproduction and Obstetrics.

[20] Sheldon I.M., Lewis G.S., LeBlanc S., Gilbert R.O. (2006). Defining Postpartum Uterine Disease in Cattle. Theriogenology.

[21] Snelling W.M., Hoff J.L., Li J.H., Kuehn L.A., Keel B.N., Lindholm-Perry A.K., Pickrell J.K. (2020). Assessment of Imputation from Low-Pass Sequencing to Predict Merit of Beef Steers. Genes.

[22] Chang C.C., Chow C.C., Tellier L.C., Vattikuti S., Purcell S.M., Lee J.J. (2015). Second-Generation PLINK: Rising to the Challenge of Larger and Richer Datasets. GigaScience.

[23] Ligges U., Mächler M. (2003). Scatterplot3d—An R Package for Visualizing Multivariate Data. J. Stat. Softw..

[24] R Core Team (2021). 2021 R: A Language and Environment for Statistical Computing 2021.

[25] Wickham H., Chang W. Package ‘Ggplot2’. Create Elegant Data Visualisations Using the Grammar of Graphics. 2016, Version 2.1. pp. 1–189. https://citeseerx.ist.psu.edu/document?repid=rep1&type=pd.

[26] Yang J., Lee S.H., Goddard M.E., Visscher P.M. (2011). GCTA: A Tool for Genome-Wide Complex Trait Analysis. Am. J. Hum. Genet..

[27] Kurz J.P., Yang Z., Weiss R.B., Wilson D.J., Rood K.A., Liu G.E., Wang Z. (2019). A Genome-Wide Association Study for Mastitis Resistance in Phenotypically Well-Characterized Holstein Dairy Cattle Using a Selective Genotyping Approach. Immunogenetics.

[28] Klein S.-L., Scheper C., May K., König S. (2020). Genetic and Nongenetic Profiling of Milk β-Hydroxybutyrate and Acetone and Their Associations with Ketosis in Holstein Cows. J. Dairy Sci..

[29] Parker Gaddis K.L., Null D.J., Cole J.B. (2016). Explorations in Genome-Wide Association Studies and Network Analyses with Dairy Cattle Fertility Traits. J. Dairy Sci..

[30] Welderufael B.G., Løvendahl P., De Koning D.-J., Janss L.L.G., Fikse W.F. (2018). Genome-Wide Association Study for Susceptibility to and Recoverability From Mastitis in Danish Holstein Cows. Front. Genet..

[31] Turner S.D. (2014). qqman: An R package for visualizing GWAS results using QQ and manhattan plots. Biorxiv.

[32] Da Y., Wang C., Wang S., Hu G. (2014). Mixed Model Methods for Genomic Prediction and Variance Component Estimation of Additive and Dominance Effects Using SNP Markers. PLoS ONE.

[33] Cunningham F., Allen J.E., Allen J., Alvarez-Jarreta J., Amode M.R., Armean I.M., Austine-Orimoloye O., Azov A.G., Barnes I., Bennett R. (2022). Ensembl 2022. Nucleic Acids Res..

[34] Huang D.W., Sherman B.T., Tan Q., Kir J., Liu D., Bryant D., Guo Y., Stephens R., Baseler M.W., Lane H.C. (2007). DAVID Bioinformatics Resources: Expanded Annotation Database and Novel Algorithms to Better Extract Biology from Large Gene Lists. Nucleic Acids Res..

[35] Hu Z.-L., Park C.A., Wu X.-L., Reecy J.M. (2013). Animal QTLdb: An Improved Database Tool for Livestock Animal QTL/Association Data Dissemination in the Post-Genome Era. Nucleic Acids Res..

[36] Galvão K.N., Bicalho R.C., Jeon S.J. (2019). Symposium Review: The Uterine Microbiome Associated with the Development of Uterine Disease in Dairy Cows. J. Dairy Sci..

[37] Horlock A.D., Piersanti R.L., Ramirez-Hernandez R., Yu F., Ma Z., Jeong K.C., Clift M.J.D., Block J., Santos J.E.P., Bromfield J.J. (2020). Uterine Infection Alters the Transcriptome of the Bovine Reproductive Tract Three Months Later. Reproduction.

[38] de Lima F.S. (2020). de Recent Advances and Future Directions for Uterine Diseases Diagnosis, Pathogenesis, and Management in Dairy Cows. Anim. Reprod..

[39] Kim D.-U., Lee S.-C., Jeong J.-K., Choi I.-S., Moon S.-H., Kang H.-G., Kim I.-H. (2016). Effects of Dystocia on the Postpartum Complications, Milk Production and Reproductive Performance in Dairy Cows. J. Vet. Clin..

[40] LeBlanc S.J. (2008). Postpartum Uterine Disease and Dairy Herd Reproductive Performance: A Review. Vet. J..

[41] Lombard J.E., Garry F.B., Tomlinson S.M., Garber L.P. (2007). Impacts of Dystocia on Health and Survival of Dairy Calves. J. Dairy Sci..

[42] Syrjälä P., Anttila M., Dillard K., Fossi M., Collin K., Nylund M., Autio T. (2007). Causes of Bovine Abortion, Stillbirth and Neonatal Death in Finland 1999–2006. Acta Vet. Scand..

[43] Fouz R., Gandoy F., Sanjuán M.L., Yus E., Diéguez F.J. (2011). Factors Associated with 56-Day Non-Return Rate in Dairy Cattle. Pesqui. Agropecuária Bras..

[44] Opsomer G., Gröhn Y.T., Hertl J., Coryn M., Deluyker H., De Kruif A. (2000). Risk Factors for Post Partum Ovarian Dysfunction in High Producing Dairy Cows in Belgium: A Field Study. Theriogenology.

[45] Ribeiro E.S., Gomes G., Greco L.F., Cerri R.L.A., Vieira-Neto A., Monteiro P.L.J., Lima F.S., Bisinotto R.S., Thatcher W.W., Santos J.E.P. (2016). Carryover Effect of Postpartum Inflammatory Diseases on Developmental Biology and Fertility in Lactating Dairy Cows. J. Dairy Sci..

[46] Dubuc J., Duffield T.F., Leslie K.E., Walton J.S., LeBlanc S.J. (2010). Risk Factors for Postpartum Uterine Diseases in Dairy Cows. J. Dairy Sci..

[47] Galvão K.N. (2013). Uterine Diseases in Dairy Cows: Understanding the Causes and Seeking Solutions. Anim. Reprod..

[48] Mu T., Hu H., Ma Y., Wen H., Yang C., Feng X., Wen W., Zhang J., Gu Y. (2022). Identifying Key Genes in Milk Fat Metabolism by Weighted Gene Co-Expression Network Analysis. Sci. Rep..

[49] Xing Y., Peng K., Yi Q., Yu D., Shi H., Yang G., Yin S. (2023). TMEM30A Is Essential for Hair Cell Polarity Maintenance in Postnatal Mouse Cochlea. Cell. Mol. Biol. Lett..

[50] Griffin S., Healey G.D., Sheldon I.M. (2018). Isoprenoids Increase Bovine Endometrial Stromal Cell Tolerance to the Cholesterol-Dependent Cytolysin from *Trueperella Pyogenes*. Biol. Reprod..

[51] He M., Zhang W., Dong Y., Wang L., Fang T., Tang W., Lv B., Chen G., Yang B., Huang P. (2017). Pro-Inflammation NF-κB Signaling Triggers a Positive Feedback via Enhancing Cholesterol Accumulation in Liver Cancer Cells. J. Exp. Clin. Cancer Res..

[52] Song N., Wang X., Gui L., Raza S.H.A., Luoreng Z., Zan L. (2017). MicroRNA-214 Regulates Immunity-Related Genes in Bovine Mammary Epithelial Cells by Targeting NFATc3 and TRAF3. Mol. Cell. Probes.

[53] Aylon Y., Oren M. (2016). The Hippo Pathway, P53 and Cholesterol. Cell Cycle.

[54] Prusinkiewicz M.A., Gameiro S.F., Ghasemi F., Dodge M.J., Zeng P.Y.F., Maekebay H., Barrett J.W., Nichols A.C., Mymryk J.S. (2020). Survival-Associated Metabolic Genes in Human Papillomavirus-Positive Head and Neck Cancers. Cancers.

[55] Brown M.S., Goldstein J.L. (1997). The SREBP Pathway: Regulation of Cholesterol Metabolism by Proteolysis of a Membrane-Bound Transcription Factor. Cell.

[56] Mavangira V., Sordillo L.M. (2018). Role of Lipid Mediators in the Regulation of Oxidative Stress and Inflammatory Responses in Dairy Cattle. Res. Vet. Sci..

[57] Strüve K., Herzog K., Magata F., Piechotta M., Shirasuna K., Miyamoto A., Bollwein H. (2013). The Effect of Metritis on Luteal Function in Dairy Cows. BMC Vet. Res..

[58] Seo E., Kang H., Choi H., Choi W., Jun H. (2019). Reactive Oxygen Species-induced Changes in Glucose and Lipid Metabolism Contribute to the Accumulation of Cholesterol in the Liver during Aging. Aging Cell.

[59] Cheng Z., Oguejiofor C., Swangchan-Uthai T., Carr S., Wathes D. (2015). Relationships between Circulating Urea Concentrations and Endometrial Function in Postpartum Dairy Cows. Animals.

[60] Geyer J., Wilke T., Petzinger E. (2006). The Solute Carrier Family SLC10: More than a Family of Bile Acid Transporters Regarding Function and Phylogenetic Relationships. Naunyn. Schmiedebergs Arch. Pharmacol..

[61] Argraves W.S., Morales C.R. (2004). Immunolocalization of Cubilin, Megalin, Apolipoprotein J, and Apolipoprotein A-I in the Uterus and Oviduct. Mol. Reprod. Dev..

[62] Hammad S.M., Stefansson S., Twal W.O., Drake C.J., Fleming P., Remaley A., Brewer H.B., Argraves W.S. (1999). Cubilin, the Endocytic Receptor for Intrinsic Factor-Vitamin B _12_ Complex, Mediates High-Density Lipoprotein Holoparticle Endocytosis. Proc. Natl. Acad. Sci. USA.

[63] Pleckaityte M. (2020). Cholesterol-Dependent Cytolysins Produced by Vaginal Bacteria: Certainties and Controversies. Front. Cell. Infect. Microbiol..

[64] Liu J., Liang Q., Wang T., Ma B., Wang X., Li P., Shaukat A., Guo X., Deng G. (2022). IFN-τ Mediated miR-26a Targeting PTEN to Activate PI3K/AKT Signalling to Alleviate the Inflammatory Damage of bEECs. Sci. Rep..

[65] Huang X., Tan J., Chen X., Liu M., Zhu H., Li W., He Z., Han J., Ma C. (2021). Akt Phosphorylation Influences Persistent Chlamydial Infection and Chlamydia-Induced Golgi Fragmentation Without Involving Rab14. Front. Cell. Infect. Microbiol..

[66] Pantazi I., Papafragkos I., Kolliniati O., Lapi I., Tsatsanis C., Vergadi E. (2022). Akt Inhibition Promotes Autophagy and Clearance of Group B Streptococcus from the Alveolar Epithelium. Pathogens.

[67] Cai C., Tang Y.-D., Zhai J., Zheng C. (2022). The RING Finger Protein Family in Health and Disease. Signal Transduct. Target. Ther..

[68] Gao Q., Srinivasan S., Boyer S.N., Wazer D.E., Band V. (1999). The E6 Oncoproteins of High-Risk Papillomaviruses Bind to a Novel Putative GAP Protein, E6TP1, and Target It for Degradation. Mol. Cell. Biol..

[69] Zou Z., Tao T., Li H., Zhu X. (2020). mTOR Signaling Pathway and mTOR Inhibitors in Cancer: Progress and Challenges. Cell Biosci..

[70] Dossou A.S., Basu A. (2019). The Emerging Roles of mTORC1 in Macromanaging Autophagy. Cancers.

[71] Ou C.-C., Hsu S.-C., Hsieh Y.-H., Tsou W.-L., Chuang T.-C., Liu J.-Y., Kao M.-C. (2008). Downregulation of HER2 by RIG1 Involves the PI3K/Akt Pathway in Ovarian Cancer Cells. Carcinogenesis.

[72] Su X., Chen D., Zhu L., Jia H., Cai J., Li P., Han B., Wang D., Li H., Fan J. (2022). SGSM2 Inhibits Thyroid Cancer Progression by Activating RAP1 and Enhancing Competitive RAS Inhibition. Cell Death Dis..

[73] Andrade G.M., Da Silveira J.C., Perrini C., Del Collado M., Gebremedhn S., Tesfaye D., Meirelles F.V., Perecin F. (2017). The Role of the PI3K-Akt Signaling Pathway in the Developmental Competence of Bovine Oocytes. PLoS ONE.

[74] Shiojima I., Walsh K. (2002). Role of Akt Signaling in Vascular Homeostasis and Angiogenesis. Circ. Res..

[75] Sipka A.S., Chandler T.L., Behling-Kelly E.L., Overton T.R., Mann S. (2020). The Effect of Ex Vivo Lipopolysaccharide Stimulation and Nutrient Availability on Transition Cow Innate Immune Cell AKT/mTOR Pathway Responsiveness. J. Dairy Sci..

[76] Wang Y., Wang X., Wang M., Zhang L., Zan L., Yang W. (2021). Bta-miR-34b Controls Milk Fat Biosynthesis via the Akt/mTOR Signaling Pathway by Targeting RAI14 in Bovine Mammary Epithelial Cells. J. Anim. Sci. Biotechnol..

[77] Wientjes Y.C., Calus M.P., Goddard M.E., Hayes B.J. (2015). Impact of QTL Properties on the Accuracy of Multi-Breed Genomic Prediction. Genet. Sel. Evol..

[78] Pimentel E.C.G., Bauersachs S., Tietze M., Simianer H., Tetens J., Thaller G., Reinhardt F., Wolf E., König S. (2011). Exploration of Relationships between Production and Fertility Traits in Dairy Cattle via Association Studies of SNPs within Candidate Genes Derived by Expression Profiling: Relationships between Production and Fertility at the Genomic Level. Anim. Genet..

[79] Brewer A., Cormican P., Lim J.J., Chapwanya A., O’Farrelly C., Meade K.G. (2020). Qualitative and Quantitative Differences in Endometrial Inflammatory Gene Expression Precede the Development of Bovine Uterine Disease. Sci. Rep..

[80] Sheldon I.M., Cronin J.G., Bromfield J.J. (2019). Tolerance and Innate Immunity Shape the Development of Postpartum Uterine Disease and the Impact of Endometritis in Dairy Cattle. Annu. Rev. Anim. Biosci..

