# Supplementary material for: Molecular Mechanisms Associated with the Development of the Metritis Complex in Dairy Cattle
Source: Genes (Basel). 2024 Mar 30;15(4):439. doi: 10.3390/genes15040439 (PMC11049392; doi:10.3390/genes15040439)
Supplement: Supplementary file 1 [file genes-15-00439-s001.zip › genes-2894617-supplementary.pdf]

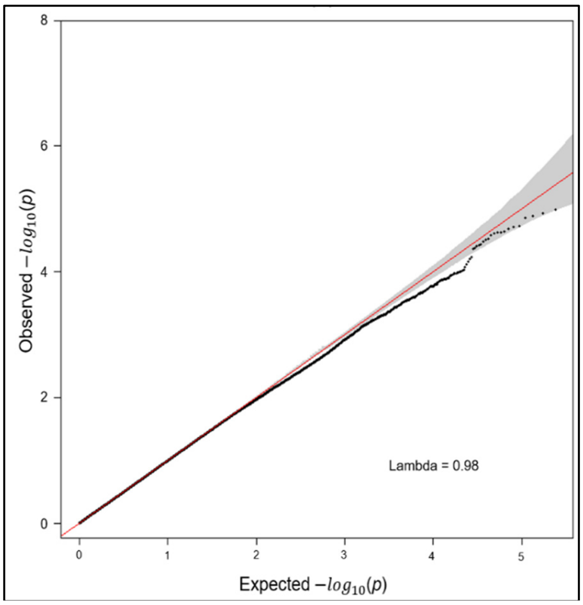

Figure S1. Quantile-Quantile (QQ) plot of the expected vs. the observed null distribution of the P-values for the association between the 624,460 SNPs and 1,967 dairy cattle across three herds diagnosed with at least one disease in the metritis complex.

Table S1. Identified single nucleotide polymorphisms (SNPs) with flanking candidate genes and associated quantitative trait loci (QTL).

BTA[1]	SNP-ID[2]	*P*-value[3]	SNP Position**	Candidate gene[4]	Linked Trait[5]	QTL ID
**12**	BovineHD1200009956	3.30E-06	33569134	*ATP8A2*	Non-return rate	QTL:5010
**10**	BovineHD1000023339	2.36E-05	81497245	*SRSF5*	Dystocia	QTL:11353
**10**	BovineHD1000023340	2.36E-05	81497728	*SRSF5*	Dystocia	QTL:11353
**10**	BTB-02098111	3.03E-05	81532849	*SLC10A1*	Dystocia	QTL:11444
**10**	BovineHD1000023344	3.05E-05	81512830	*SLC10A1*	Dystocia	QTL:11444
**10**	BovineHD1000023769	3.31E-05	83080675	*SIPA1L1*	Stillbirth	QTL:11445
**10**	BTA-77307-no-rs	3.96E-05	81502058	*SRSF5*	Dystocia	QTL:11353
**17**	BovineHD1700021176	4.33E-05	70481417	*DEPDC5*	Non-return rate	QTL:141715
**10**	BovineHD1000023497	5.78E-05	82066559	*COX16*	Stillbirth	QTL:11445
**15**	BovineHD1500002327	7.55E-05	8809053	*CNTN5*	Non-return rate	QTL:3571
**11**	BovineHD0500026389	8.59E-05	92476148	*DAB2IP*	Stillbirth	QTL:126867
**10**	BovineHD1000025233	9.64E-05	87632404	*ESRRB*	Placental Retention	QTL:11316
**21**	BovineHD2100020220	1.03E-04	67602910	*TRAF3*	____	____
				AMN	____	____
**8**	BovineHD0800007389	1.05E-04	24598682	*SLC24A2*	Stillbirth	QTL:11443
**10**	BovineHD1000025221	1.13E-04	87619311	*ESRRB*	Placental Retention	QTL:11316
**10**	BovineHD1000023418	1.15E-04	81811346	*SLC8A3*	Dystocia	QTL:11353
**10**	BovineHD1000025236	1.27E-04	87637414	*ESRRB*	Placental Retention	QTL:11316
**10**	BovineHD1000025235	1.27E-04	87634538	*ESRRB*	Placental Retention	QTL:11316
**11**	BovineHD1100026876	1.28E-04	92476478	*DAB2IP*	Stillbirth	QTL:126867

[1] BTA =Bos taurus autosome

[2] SNP = Single nucleotide polymorphism; SNPs listed in ascending order with respect to P-value

[3] Genome-wide significance threshold

[4] Nearest gene 10k base pair downstream/upstream relative to the SNP

[5] Previously associated trait; Details obtained from the Animal QTLdb for cattle [36]

**27**	BovineHD2700007980	1.31E-04	29252315	*RNF122*	Dystocia	QTL:11393
**17**	BovineHD1700019549	1.33E-04	65237605	*KIAA1671*	Calving ease	QTL:11051
**11**	BovineHD1100026874	1.33E-04	92473780	*DAB2IP*	Stillbirth	QTL:126867
**11**	BovineHD1100026881	1.37E-04	92481234	*DAB2IP*	Stillbirth	QTL:126867
**10**	BovineHD1000025230	1.39E-04	87629150	*ESRRB*	Placental Retention	QTL:11316
**11**	BovineHD1100026880	1.59E-04	92480213	*DAB2IP*	Stillbirth	QTL:126867
**4**	BovineHD0400018341	1.71E-04	66416290	*SCRN1*	Calving Ease	QTL:10713
				WIPF3	Calving Ease	QTL:10713
**10**	BovineHD1000025232	1.73E-04	87631188	*ESRRB*	Placental Retention	QTL:11316
**10**	BovineHD1000025229	1.73E-04	87628421	*ESRRB*	Placental Retention	QTL:11316
**10**	BovineHD1000025231	1.75E-04	87630598	*ESRRB*	Placental Retention	QTL:11316
**8**	BovineHD0800007387	1.76E-04	24596562	*SLC24A2*	Stillbirth	QTL:11443
**11**	BovineHD1100026875	1.86E-04	92475887	*DAB2IP*	Stillbirth	QTL:126867
**11**	BovineHD1100026877	1.95E-04	92477673	*DAB2IP*	Stillbirth	QTL:126867
**10**	BovineHD1000025228	2.09E-04	87627458	*ESRRB*	Placental Retention	QTL:11316
**10**	BovineHD1000023822	2.15E-04	83245103	*SIPA1L1*	Stillbirth	QTL:11445
**25**	BovineHD2500009545	2.20E-04	34170987	*POR*	Calving ease	QTL:4674
**8**	BovineHD0800007390	2.26E-04	24599571	*SLC24A2*	Stillbirth	QTL:11443
**11**	BovineHD1100005785	2.38E-04	18909925	*CRIM1*	-	-
**10**	BovineHD1000025245	2.44E-04	87650109	*ESRRB*	Placental Retention	QTL:11316
**18**	BovineHD1800003585	2.47E-04	10026821	*CDH13*	Dystocia	QTL:2707

Table S2. Enriched gene ontology (GO) biological process (BP) terms for the metritis complex in Jersey and Holstein dairy cattle.

GO-ID[6]	Term	p-value	Gene(s) Assoc.[7]
**GO:0098703**	Calcium Ion Import Across Plasma Membrane	0.015	*SLC8A3, SLC24A2*
**GO:0032088**	Negative Regulation of NF-KappaB Transcription Factor Activity	0.062	*DAB2IP, TRAF3*
**GO:0043087**	Regulation of GTPase Activity	0.065	*DAB2IP, SIPA1L1*
**GO:0046330**	Positive Regulation of JNK Cascade	0.076	*DAB2IP, TRAF3*
**GO:0043065**	Positive Regulation of Apoptotic Process	0.184	*DAB2IP, RNF122*
**GO:0008285**	Negative Regulation of Cell Proliferation	0.214	*CDH13, DAB2IP*
**GO:0045944**	Positive Regulation of Transcription from RNA Polymerase II Promoter	0.510	*CDH13, DAB2IP*
**GO:1900744**	Regulation of P38Mapk Cascade	1.000	*DAB2IP*
**GO:0070317**	Negative Regulation of G0 to G1 Transition	1.000	*DAB2IP*
**GO:0030032**	Lamellipodium Assembly	1.000	*CDH13*
**GO:0048661**	Positive Regulation of Smooth Muscle Cell Proliferation	1.000	*CDH13*
**GO:0050680**	Negative Regulation of Epithelial Cell Proliferation	1.000	*DAB2IP*
**GO:0043124**	Negative Regulation of I-KappaB Kinase/NF-KappaB Signaling	1.000	*DAB2IP*
**GO:0007162**	Negative Regulation of Cell Adhesion	1.000	*CDH13*
**GO:0043542**	Endothelial Cell Migration	1.000	*CDH13*
**GO:0014067**	Negative Regulation of Phosphatidylinositol 3-Kinase Signaling	1.000	*DAB2IP*
**GO:0070588**	Calcium Ion Transmembrane Transport	1.000	*SLC24A2*

[6] GO-ID = Gene Ontology Identification Number

[7] Gene(s) Assoc. = Genes linked to significant genetic variants in the metritis complex GWAS *in-silico* functional analysis

**GO:0030335**	Positive Regulation of Cell Migration	1.000	*CDH13*
**GO:0010917**	Negative Regulation of Mitochondrial Membrane Potential	1.000	*RNF122*
**GO:0007156**	Homophilic Cell Adhesion Via Plasma Membrane Adhesion Molecules	1.000	*CDH13*
**GO:0051865**	Protein Autoubiquitination	1.000	*RNF122*
**GO:0090346**	Cellular Organofluorine Metabolic Process	1.000	*POR*
**GO:0036324**	Vascular Endothelial Growth Factor Receptor-2 Signaling Pathway	1.000	*DAB2IP*
**GO:0050688**	Regulation of Defense Response to Virus	1.000	*TRAF3*
**GO:0070534**	Protein K63-Linked Ubiquitination	1.000	*TRAF3*
**GO:0034260**	Negative Regulation of GTPase Activity	1.000	*DAB2IP*
**GO:0007588**	Excretion	1.000	*AMN*
**GO:0034198**	Cellular Response to Amino Acid Starvation	1.000	*DEPDC5*
**GO:0007613**	Memory	1.000	*SLC24A2*
**GO:0043161**	Proteasome-Mediated Ubiquitin-Dependent Protein Catabolic Process	1.000	*RNF122*
**GO:0048814**	Regulation of Dendrite Morphogenesis	1.000	*SIPA1L1*
**GO:0015914**	Phospholipid Transport	1.000	*ATP8A2*
**GO:0050927**	Positive Regulation of Positive Chemotaxis	1.000	*CDH13*
**GO:0000122**	Negative Regulation of Transcription from RNA Polymerase II Promoter	1.000	*DAB2IP*
**GO:0050770**	Regulation of Axonogenesis	1.000	*SIPA1L1*
**GO:0060292**	Long Term Synaptic Depression	1.000	*SLC24A2*
**GO:0043122**	Regulation of I-KappaB Kinase/NF-KappaB Signaling	1.000	*TRAF3*
**GO:0045332**	Phospholipid Translocation	1.000	*ATP8A2*
**GO:0007601**	Visual Perception	1.000	*SLC24A2*
**GO:0021814**	Cell Motility Involved in Cerebral Cortex Radial Glia Guided Migration	1.000	*DAB2IP*

**GO:0008625**	Extrinsic Apoptotic Signaling Pathway Via Death Domain Receptors	1.000	*DAB2IP*
**GO:1900006**	Positive Regulation of Dendrite Development	1.000	*DAB2IP*
**GO:0035556**	Intracellular Signal Transduction	1.000	*DEPDC5*
**GO:0030514**	Negative Regulation of Bmp Signaling Pathway	1.000	*CRIM1*
**GO:0030048**	Actin Filament-Based Movement	1.000	*WIPF3*
**GO:0090630**	Activation of GTPase Activity	1.000	*SIPA1L1*
**GO:0035148**	Tube Formation	1.000	*DAB2IP*
**GO:0030948**	Negative Regulation of Vascular Endothelial Growth Factor Receptor Signaling Pathway	1.000	*DAB2IP*
**GO:0030001**	Metal Ion Transport	1.000	*SLC8A3*
**GO:0051056**	Regulation of Small GTPase Mediated Signal Transduction	1.000	*SIPA1L1*
**GO:0006357**	Regulation of Transcription from RNA Polymerase II Promoter	1.000	*ESRRB*
**GO:0098742**	Cell-Cell Adhesion Via Plasma-Membrane Adhesion Molecules	1.000	*CDH13*
**GO:0010506**	Regulation of Autophagy	1.000	*DEPDC5*
**GO:0016339**	Calcium-Dependent Cell-Cell Adhesion Via Plasma Membrane Cell Adhesion Molecules	1.000	*CDH13*
**GO:0045668**	Negative Regulation of Osteoblast Differentiation	1.000	*CRIM1*
**GO:0048147**	Negative Regulation of Fibroblast Proliferation	1.000	*DAB2IP*
**GO:0043616**	Keratinocyte Proliferation	1.000	*CDH13*
**GO:0010596**	Negative Regulation of Endothelial Cell Migration	1.000	*DAB2IP*
**GO:0000398**	MRNA Splicing, Via Spliceosome	1.000	*SRSF5*
**GO:0043407**	Negative Regulation of Map Kinase Activity	1.000	*DAB2IP*
**GO:0070373**	Negative Regulation of Erk1 and Erk2 Cascade	1.000	*DAB2IP*
**GO:2001224**	Positive Regulation of Neuron Migration	1.000	*DAB2IP*
**GO:0007154**	Cell Communication	1.000	*SLC8A3*

**GO:0048013**	Ephrin Receptor Signaling Pathway	1.000	*SIPA1L1*
**GO:0030162**	Regulation of Proteolysis	1.000	*TRAF3*
**GO:0044257**	Cellular Protein Catabolic Process	1.000	*DAB2IP*
**GO:0090129**	Positive Regulation of Synapse Maturation	1.000	*DAB2IP*
**GO:0021819**	Layer Formation in Cerebral Cortex	1.000	*DAB2IP*
**GO:0001817**	Regulation of Cytokine Production	1.000	*TRAF3*
**GO:0060291**	Long-Term Synaptic Potentiation	1.000	*SLC24A2*
**GO:0048666**	Neuron Development	1.000	*ATP8A2*
**GO:0030100**	Regulation of Endocytosis	1.000	*CDH13*
**GO:0031532**	Actin Cytoskeleton Reorganization	1.000	*SIPA1L1*
**GO:0038026**	Reelin-Mediated Signaling Pathway	1.000	*DAB2IP*
**GO:0007266**	Rho Protein Signal Transduction	1.000	*CDH13*
**GO:0031334**	Positive Regulation of Protein Complex Assembly	1.000	*DAB2IP*
**GO:0008063**	Toll Signaling Pathway	1.000	*TRAF3*
**GO:0032770**	Positive Regulation of Monooxygenase Activity	1.000	*POR*
**GO:0007252**	I-KappaB Phosphorylation	1.000	*DAB2IP*
**GO:1901800**	Positive Regulation of Proteasomal Protein Catabolic Process	1.000	*DAB2IP*
**GO:0043553**	Negative Regulation of Phosphatidylinositol 3-Kinase Activity	1.000	*DAB2IP*
**GO:0045087**	Innate Immune Response	1.000	*TRAF3*
**GO:0002224**	Toll-Like Receptor Signaling Pathway	1.000	*TRAF3*
**GO:0016525**	Negative Regulation of Angiogenesis	1.000	*DAB2IP*
**GO:0071222**	Cellular Response to Lipopolysaccharide	1.000	*DAB2IP*
**GO:0009725**	Response to Hormone	1.000	*POR*
**GO:0033617**	Mitochondrial Respiratory Chain Complex Iv Assembly	1.000	*COX16*
**GO:0006508**	Proteolysis	1.000	*SCRN1*
**GO:0034144**	Negative Regulation of Toll-Like Receptor 4 Signaling Path-	1.000	*DAB2IP*

	way		
**GO:1903363**	Negative Regulation of Cellular Protein Catabolic Process	1.000	*DAB2IP*
**GO:0006874**	Cellular Calcium Ion Homeostasis	1.000	*SLC24A2*
**GO:0006887**	Exocytosis	1.000	*SCRN1*
**GO:0090090**	Negative Regulation of Canonical WNT Signaling Pathway	1.000	*DAB2IP*
**GO:0001954**	Positive Regulation of Cell-Matrix Adhesion	1.000	*CDH13*
**GO:0033209**	Tumor Necrosis Factor-Mediated Signaling Pathway	1.000	*TRAF3*
**GO:0032007**	Negative Regulation of Tor Signaling	1.000	*DEPDC5*
**GO:0015889**	Cobalamin Transport	1.000	*AMN*
**GO:0035924**	Cellular Response to Vascular Endothelial Growth Factor Stimulus	1.000	*DAB2IP*
**GO:0055096**	Low-Density Lipoprotein Particle Mediated Signaling	1.000	*CDH13*
**GO:0070059**	Intrinsic Apoptotic Signaling Pathway in Response to Endoplasmic Reticulum Stress	1.000	*DAB2IP*
**GO:0015721**	Bile Acid and Bile Salt Transport	1.000	*SLC10A1*
**GO:0050850**	Positive Regulation of Calcium-Mediated Signaling	1.000	*CDH13*
**GO:0051668**	Localization Within Membrane	1.000	*CDH13*
**GO:0043507**	Positive Regulation of Jun Kinase Activity	1.000	*DAB2IP*
**GO:0001938**	Positive Regulation of Endothelial Cell Proliferation	1.000	*CDH13*
**GO:0006898**	Receptor-Mediated Endocytosis	1.000	*AMN*
**GO:0007612**	Learning	1.000	*SLC24A2*
**GO:0043001**	Golgi to Plasma Membrane Protein Transport	1.000	*AMN*
**GO:0022900**	Electron Transport Chain	1.000	*POR*
**GO:0008104**	Protein Localization	1.000	*AMN*
**GO:0071347**	Cellular Response to Interleukin-1	1.000	*DAB2IP*
**GO:2001235**	Positive Regulation of Apoptotic Signaling Pathway	1.000	*DAB2IP*
**GO:0032648**	Regulation of Interferon-Beta Production	1.000	*TRAF3*

**GO:0042981**	Regulation of Apoptotic Process	1.000	*TRAF3*
**GO:0015031**	Protein Transport	1.000	*AMN*
**GO:0035725**	Sodium Ion Transmembrane Transport	1.000	*SLC8A3*
**GO:0010719**	Negative Regulation of Epithelial to Mesenchymal Transition	1.000	*DAB2IP*
**GO:0002040**	Sprouting Angiogenesis	1.000	*CDH13*
**GO:0016601**	RAC Protein Signal Transduction	1.000	*CDH13*
**GO:0048812**	Neuron Projection Morphogenesis	1.000	*DAB2IP*
**GO:0042058**	Regulation of Epidermal Growth Factor Receptor Signaling Pathway	1.000	*CDH13*
**GO:0071356**	Cellular Response to Tumor Necrosis Factor	1.000	*DAB2IP*
**GO:1900747**	Negative Regulation of Vascular Endothelial Growth Factor Signaling Pathway	1.000	*DAB2IP*

Table S3. Enriched gene ontology (GO) cellular component (CC) terms for the metritis complex in Jersey and Holstein dairy cattle.

GO-ID[8]	Term	p-value	Gene(s) Assoc.[9]
**GO:0030139**	Endocytic Vesicle	0.035	*DAB2IP, AMN*
**GO:0005737**	Cytoplasm	0.203	*CDH13, DEPDC5, RNF122, SIPA1L1, SCRN1, TRAF3*
**GO:0016021**	Integral Component of Membrane	0.343	*COX16, ATP8A2, POR, RNF122, CRIM1, SLC10A1, AMN, SLC8A3*
**GO:0005887**	Integral Component of Plasma Membrane	0.529	*SLC8A3, SLC24A2*
**GO:0005886**	Plasma Membrane	0.645	*ATP8A2, CDH13, SIPA1L1, AMN, SLC8A3*
**GO:0005615**	Extracellular Space	0.690	*CDH13, AMN*
**GO:0005829**	Cytosol	0.925	*SRSF5, POR*
**GO:0005634**	Nucleus	1.000	*ESRRB*
**GO:0009897**	External Side of Plasma Membrane	1.000	*CDH13*
**GO:0043005**	Neuron Projection	1.000	*CDH13*
**GO:1990032**	Parallel Fiber	1.000	*DAB2IP*
**GO:0031965**	Nuclear Membrane	1.000	*SCRN1*
**GO:0005884**	Actin Filament	1.000	*WIPF3*
**GO:0005730**	Nucleolus	1.000	*SRSF5*
**GO:0005901**	Caveola	1.000	*CDH13*
**GO:0010008**	Endosome Membrane	1.000	*ATP8A2*
**GO:0042383**	Sarcolemma	1.000	*SLC8A3*
**GO:0098794**	Post synapse	1.000	*SLC8A3*
**GO:0097381**	Photoreceptor Disc Membrane	1.000	*ATP8A2*
**GO:0032809**	Neuronal Cell Body Membrane	1.000	*DAB2IP*
**GO:0012505**	Endomembrane System	1.000	*RNF122*
**GO:0035631**	Cd40 Receptor Complex	1.000	*TRAF3*
**GO:0015629**	Actin Cytoskeleton	1.000	*SIPA1L1*
**GO:0031305**	Integral Component of Mitochondrial Inner Membrane	1.000	*COX16*

[8]GO-ID = Gene Ontology Identification Number

[9]Gene(s) Assoc. = Genes linked to significant genetic variants in the metritis complex GWAS in-silico functional analysis

**GO:0031225**	Anchored Component of Membrane	1.000	*CDH13*
**GO:0005789**	Endoplasmic Reticulum Membrane	1.000	*POR*
**GO:0016342**	Catenin Complex	1.000	*CDH13*
**GO:0044301**	Climbing Fiber	1.000	*DAB2IP*
**GO:0043235**	Receptor Complex	1.000	*AMN*
**GO:0016020**	Membrane	1.000	*SLC8A3*
**GO:0005743**	Mitochondrial Inner Membrane	1.000	*COX16*
**GO:1990597**	Aip1-Ire1 Complex	1.000	*DAB2IP*
**GO:0030424**	Axon	1.000	*SLC8A3*
**GO:0005765**	Lysosomal Membrane	1.000	*DEPDC5*
**GO:0016324**	Apical Plasma Membrane	1.000	*AMN*
**GO:0009898**	Cytoplasmic Side of Plasma Membrane	1.000	*TRAF3*
**GO:0005794**	Golgi Apparatus	1.000	*ATP8A2*
**GO:0001917**	Photoreceptor Inner Segment	1.000	*ATP8A2*
**GO:1990531**	Lem3P-Dnf1P Complex	1.000	*ATP8A2*
**GO:0016607**	Nuclear Speck	1.000	*SRSF5*
**GO:1990130**	Iml1 Complex	1.000	*DEPDC5*
**GO:0044300**	Cerebellar Mossy Fiber	1.000	*DAB2IP*
**GO:0000139**	Golgi Membrane	1.000	*ATP8A2*
**GO:0005768**	Endosome	1.000	*ATP8A2*
**GO:0005576**	Extracellular Region	1.000	*CRIM1*
**GO:0005654**	Nucleoplasm	1.000	*ATP8A2*
**GO:0048471**	Perinuclear Region of Cytoplasm	1.000	*SLC8A3*

Table S4. Enriched gene ontology (GO) molecular function (MF) terms for the metritis complex in Jersey and Holstein dairy cattle.

GO-ID[10]	Term	*P*-value	Gene(s) Assoc.[11]
**GO:0005096**	GTPase Activator Activity	0.011	*DAB2IP, DEPDC5, SI-PA1L1*
**GO:0042803**	Protein Homodimerization Activity	0.384	*CDH13, DAB2IP*
**GO:0008270**	Zinc Ion Binding	0.450	*ESRRB, TRAF3*
**GO:0046872**	Metal Ion Binding	0.749	*RNF122, SLC8A3*
**GO:0031625**	Ubiquitin Protein Ligase Binding	1.000	*TRAF3*
**GO:0015491**	Cation: Cation Antiporter Activity	1.000	*SLC8A3*
**GO:0004879**	RNA Polymerase II Transcription Factor Activity, Ligand-Activated Sequence-Specific DNA Binding	1.000	*ESRRB*
**GO:0042802**	Identical Protein Binding	1.000	*TRAF3*
**GO:0005524**	ATP Binding	1.000	*ATP8A2*
**GO:0019901**	Protein Kinase Binding	1.000	*TRAF3*
**GO:0003729**	MRNA Binding	1.000	*SRSF5*
**GO:0005102**	Receptor Binding	1.000	*AMN*
**GO:0045296**	Cadherin Binding	1.000	*CDH13*
**GO:0090555**	Phosphatidylethanolamine-Translocating ATPase Activity	1.000	*ATP8A2*
**GO:0008273**	Calcium, Potassium: Sodium Antiporter Activity	1.000	*SLC24A2*
**GO:0005516**	Calmodulin Binding	1.000	*SLC8A3*
**GO:0000978**	RNA Polymerase II Core Promoter Proximal Region Sequence-Specific DNA Binding	1.000	*ESRRB*
**GO:0005262**	Calcium Channel Activity	1.000	*SLC24A2*
**GO:0003723**	RNA Binding	1.000	*SRSF5*
**GO:0043539**	Protein Serine/Threonine Kinase Activator Activity	1.000	*DAB2IP*
**GO:0031996**	Thioesterase Binding	1.000	*TRAF3*
**GO:0003707**	Steroid Hormone Receptor Activity	1.000	*ESRRB*
**GO:0051721**	Protein Phosphatase 2A Binding	1.000	*DAB2IP*
**GO:0035591**	Signaling Adaptor Activity	1.000	*DAB2IP*

[10]GO-ID = Gene Ontology Identification Number

[11]Gene(s) Assoc. = Genes linked to significant genetic variants in the metritis complex GWAS in-silico functional analysis

**GO:0000287**	Magnesium Ion Binding	1.000	*ATP8A2*
**GO:0090556**	Phosphatidylserine-Translocating ATPase Activity	1.000	*ATP8A2*
**GO:0016887**	ATPase Activity	1.000	*ATP8A2*
**GO:0005496**	Steroid Binding	1.000	*ESRRB*
**GO:0019903**	Protein Phosphatase Binding	1.000	*TRAF3*
**GO:0050661**	NADP Binding	1.000	*POR*
**GO:0017124**	Sh3 Domain Binding	1.000	*DAB2IP*
**GO:0008508**	Bile Acid: Sodium Symporter Activity	1.000	*SLC10A1*
**GO:0031435**	Mitogen-Activated Protein Kinase Kinase Kinase Binding	1.000	*DAB2IP*
**GO:0070004**	Cysteine-Type Exopeptidase Activity	1.000	*SCRN1*
**GO:0032266**	Phosphatidylinositol-3-Phosphate Binding	1.000	*DAB2IP*
**GO:0003958**	NADPH-Hemoprotein Reductase Activity	1.000	*POR*
**GO:0005164**	Tumor Necrosis Factor Receptor Binding	1.000	*TRAF3*
**GO:0043565**	Sequence-Specific DNA Binding	1.000	*ESRRB*
**GO:0070273**	Phosphatidylinositol-4-Phosphate Binding	1.000	*DAB2IP*
**GO:0050660**	Flavin Adenine Dinucleotide Binding	1.000	*POR*
**GO:0030169**	Low-Density Lipoprotein Particle Binding	1.000	*CDH13*
**GO:0003700**	Transcription Factor Activity, Sequence-Specific DNA Binding	1.000	*ESRRB*
**GO:0016805**	Dipeptidase Activity	1.000	*SCRN1*
**GO:0071889**	14-3-3 Protein Binding	1.000	*DAB2IP*
**GO:0031434**	Mitogen-Activated Protein Kinase Kinase Binding	1.000	*DAB2IP*
**GO:0046875**	Ephrin Receptor Binding	1.000	*SIPA1L1*
**GO:0030165**	PDZ Domain Binding	1.000	*CRIM1*
**GO:0005123**	Death Receptor Binding	1.000	*DAB2IP*
**GO:0010181**	FMN Binding	1.000	*POR*
**GO:0005520**	Insulin-Like Growth Factor Binding	1.000	*CRIM1*
**GO:0043184**	Vascular Endothelial Growth Factor Receptor 2 Binding	1.000	*DAB2IP*
**GO:0004867**	Serine-Type Endopeptidase Inhibitor Activity	1.000	*CRIM1*
**GO:0044877**	Macromolecular Complex Binding	1.000	*DAB2IP*
**GO:0005432**	Calcium: Sodium Antiporter Activity	1.000	*SLC8A3*
**GO:0005509**	Calcium Ion Binding	1.000	*CDH13*
**GO:0016491**	Oxidoreductase Activity	1.000	*POR*

**GO:0036312**	Phosphatidylinositol 3-Kinase Regulatory Subunit Binding	1.000	*DAB2IP*
**GO:0055100**	Adiponectin Binding	1.000	*CDH13*
**GO:0061630**	Ubiquitin Protein Ligase Activity	1.000	*RNF122*

Table S5. Enriched Kyoto Encyclopedia of Genes and Genomes (KEGG) pathways for the metritis complex in Jersey and Holstein dairy cattle.

Pathway ID[12]	Term	p-value	Gene(s) Assoc. [13]
**bta04668**	TNF Signaling Pathway	0.117	*DAB2IP, TRAF3*
**bta05161**	Hepatitis B	0.163	*SLC10A1, TRAF3*
**bta05168**	Herpes Simplex Virus 1 Infection	0.346	*SRSF5, TRAF3*
**bta04144**	Endocytosis	1.000	*WIPF3*
**bta04064**	NF-KappaB Signaling Pathway	1.000	*TRAF3*
**bta05412**	Arrhythmogenic Right Ventricular Cardiomyopathy	1.000	*SLC8A3*
**bta05160**	Hepatitis C	1.000	*TRAF3*
**bta04978**	Mineral Absorption	1.000	*SLC8A3*
**bta04620**	Toll-Like Receptor Signaling Pathway	1.000	*TRAF3*
**bta04714**	Thermogenesis	1.000	*COX16*
**bta04260**	Cardiac Muscle Contraction	1.000	*SLC8A3*
**bta04210**	Apoptosis	1.000	*DAB2IP*
**bta05165**	Human Papillomavirus Infection	1.000	*TRAF3*
**bta04622**	Rig-I-Like Receptor Signaling Pathway	1.000	*TRAF3*
**bta04020**	Calcium Signaling Pathway	1.000	*SLC8A3*
**bta05162**	Measles	1.000	*TRAF3*
**bta05200**	Pathways In Cancer	1.000	*TRAF3*
**bta04550**	Signaling Pathways Regulating Pluripotency of Stem Cells	1.000	*ESRRB*
**bta04976**	Bile Secretion	1.000	*SLC10A1*
**bta04621**	Nod-Like Receptor Signaling Pathway	1.000	*TRAF3*
**bta04022**	cGMP-Pkg Signaling Pathway	1.000	*SLC8A3*
**bta04740**	Olfactory Transduction	1.000	*SLC8A3*

[12] Pathway ID = KEGG Pathway Identification Number

[13] Gene(s) Assoc. = Genes linked to significant genetic variants in the metritis complex GWAS in-silico functional analysis

**bta05164**	Influenza A	1.000	*TRAF3*
**bta04974**	Protein Digestion and Absorption	1.000	*SLC8A3*
**bta04261**	Adrenergic Signaling in Cardiomyocytes	1.000	*SLC8A3*
**bta04371**	Apelin Signaling Pathway	1.000	*SLC8A3*
**bta05171**	Coronavirus Disease - Covid-19	1.000	*TRAF3*
**bta05222**	Small Cell Lung Cancer	1.000	*TRAF3*
**bta03040**	Spliceosome	1.000	*SRSF5*
**bta04150**	mTOR Signaling Pathway	1.000	*DEPDC5*
**bta05417**	Lipid and Atherosclerosis	1.000	*TRAF3*
**bta04657**	Il-17 Signaling Pathway	1.000	*TRAF3*
**bta05410**	Hypertrophic Cardiomyopathy	1.000	*SLC8A3*
**bta05414**	Dilated Cardiomyopathy	1.000	*SLC8A3*
**bta05135**	Yersinia Infection	1.000	*WIPF3*
**bta04015**	Rap1 Signaling Pathway	1.000	*SIPA1L1*
**bta05169**	Epstein-Barr Virus Infection	1.000	*TRAF3*
**bta05203**	Viral Carcinogenesis	1.000	*TRAF3*
**bta04936**	Alcoholic Liver Disease	1.000	*TRAF3*
**bta04961**	Endocrine and Other Factor-Regulated Calcium Reabsorption	1.000	*SLC8A3*
**bta05167**	Kaposi Sarcoma-Associated Herpesvirus Infection	1.000	*TRAF3*
